# Right frontal cingulate cortex mediates the effect of prenatal complications on youth internalizing behaviors

**DOI:** 10.1038/s41380-024-02475-y

**Published:** 2024-02-20

**Authors:** Eleonora Maggioni, Alessandro Pigoni, Elisa Fontana, Giuseppe Delvecchio, Carolina Bonivento, Valentina Bianchi, Maddalena Mauri, Monica Bellina, Rossano Girometti, Nivedita Agarwal, Maria Nobile, Paolo Brambilla

**Affiliations:** 1 Department of Electronics Information and Bioengineering, Politecnico di Milano, Milano, Italy; 2 Department of Neurosciences and Mental Health, Fondazione IRCCS Ca’ Granda Ospedale Maggiore Policlinico, Milan, Italy; 3 Social and Affective Neuroscience Group, MoMiLab, IMT School for Advanced Studies Lucca, Lucca, Italy; 4 Department of Pathophysiology and Transplantation, University of Milan, Milan, Italy; 5 Scientific Institute IRCCS “Eugenio Medea”, Pasian di Prato (Ud), Italy; 6 Child and Adolescent Psychiatry Unit, Scientific Institute IRCCS “Eugenio Medea”, Bosisio Parini (Lc), Italy; 7 Institute of Radiology, Department of Medicine (DMED), University of Udine, Udine, Italy; 8 University Hospital S. Maria Della Misericordia, Azienda Sanitaria Universitaria Friuli Centrale (ASUFC), Udine, Italy; 9 Neuroimaging Unit, Scientific Institute IRCCS “Eugenio Medea”, Bosisio Parini (Lc), Italy

**Keywords:** Predictive markers, Prognostic markers

## Abstract

Prenatal and perinatal complications represent well-known risk factors for the future development of psychiatric disorders. Such influence might become manifested during childhood and adolescence, as key periods for brain and behavioral changes. Internalizing and externalizing behaviors in adolescence have been associated with the risk of psychiatric onset later in life. Both brain morphology and behavior seem to be affected by obstetric complications, but a clear link among these three aspects is missing. Here, we aimed at analyzing the association between prenatal and perinatal complications, behavioral issues, and brain volumes in a group of children and adolescents. Eighty-two children and adolescents with emotional-behavioral problems underwent clinical and 3 T brain magnetic resonance imaging (MRI) assessments. The former included information on behavior, through the Child Behavior Checklist/6-18 (CBCL/6-18), and on the occurrence of obstetric complications. The relationships between clinical and gray matter volume (GMV) measures were investigated through multiple generalized linear models and mediation models. We found a mutual link between prenatal complications, GMV alterations in the frontal gyrus, and withdrawn problems. Specifically, complications during pregnancy were associated with higher CBCL/6-18 withdrawn scores and GMV reductions in the right superior frontal gyrus and anterior cingulate cortex. Finally, a mediation effect of these GMV measures on the association between prenatal complications and the withdrawn dimension was identified. Our findings suggest a key role of obstetric complications in affecting brain structure and behavior. For the first time, a mediator role of frontal GMV in the relationship between prenatal complications and internalizing symptoms was suggested. Once replicated on independent cohorts, this evidence will have relevant implications for planning preventive interventions.

## Introduction

In recent years, an interdependence between family environment, including parent psychopathology, and offspring outcomes has been suggested [1]. Specifically, prenatal and perinatal complications represent well-known risk factors for developing psychiatric disorders later in life [2–5]. Indeed, birth complications were found to be associated with a higher risk of developing schizophrenia, depression, and affective disorders [6–15].

However, the majority of studies on this topic have been performed in a retrospective fashion on adult samples [8, 16, 17]. Thus, the available evidence prevents from clearly separating prenatal and perinatal complications from other causes, since the risk for psychiatric disorders is also related to the complex interplay between genes and postnatal environmental factors, such as maternal stress, maltreatment, parenting behavior during childhood, living in a highly urbanized environment, and experiencing social exclusion [1, 18–20]. Although it is difficult to completely remove the influence of postnatal environmental factors on behavior, studying the impact of obstetric complications in the earlier stages of development can limit the confounding effects of environment. Therefore, in recent years, the scientific community has driven its attention towards the study of children, adolescents, as well as twins [21–24], with the final aim of clarifying the specific role of obstetric risk factors in the development of psychiatric illnesses.

Indeed, childhood and adolescence represent key periods for brain and behavioral changes that often concur with the emergence of early mental health symptoms [25], and obstetric complications proved to influence both brain morphology and behavior in studies conducted in these age ranges [26, 27].

As concerns the influence on brain development, a twin study [28] showed a significant association between low birth weight and reduced gray matter volume (GMV) and white matter volume (WMV) in the superior frontal gyrus and thalamus in adolescents. Similarly, maternal stress during pregnancy was found associated with bilateral posterior parietal cortex alterations in adolescents [26].

Furthermore, behaviors and mental states in these key periods of life are often regarded as relevant factors for later development of psychiatric disorders [29–32], in particular internalizing and externalizing behaviors [33], which comprise two different states of emotional and social problems [29]. More specifically, internalizing problems encompass inwardly focused behaviors, such as depression, anxiety, and somatic symptoms, while externalizing problems refer to outwardly focused behaviors, such as hyperactivity, aggressive attitude, disruptive conduct, and substance use [32]. As a consequence, young individuals with persistent internalizing and externalizing behaviors are more likely to develop a mental health disorder in adulthood [30, 31], with internalizing symptoms increasing the risk for unipolar depressive disorders [34], panic attacks [35], and anxiety disorders [36], while externalizing symptoms increasing the susceptibility to substance abuse [37] and aggression and violence [38, 39]. Standardized tests like the Child Behavior Checklist (CBCL) are employed to identify such behaviors and can help predict DSM diagnoses [33, 40, 41].

Interestingly, both behaviors can be affected by pre- and peri-natal complications. Indeed, complications like heavy bleeding, hypertension, or excessive fluid retention represent risk factors for childhood anxiety disorders [27]. Furthermore, maternal smoking during pregnancy may predict internalizing and externalizing psychopathology in offspring [42, 43]. On the other hand, internalizing and externalizing behaviors have been linked to abnormal brain structure in adolescents [44]. Specifically, internalizing behaviors seem to be related to reduced GMV in limbic/paralimbic areas [45, 46], while externalized-based disorders such as attention deficit hyperactivity disorder seem to be characterized by decreased volumes in the whole brain and in specific cortical and subcortical regions, namely the prefrontal cortex and limbic structures [47, 48].

Despite its relevance, the research investigating the association between early risk factors, brain development, and behavioral phenotypes is still at its outset. Although the pairwise associations between obstetric complications, altered brain morphometry, and behavioral problems have been investigated, albeit not in detail, no study explored the complex and mutual interplay among these three components.

In the present study, we aimed at examining the impact of prenatal and perinatal complications on brain structure and behavioral traits in children and adolescents who took part in the multicentric GENESIS project [33, 49]. We also aimed at assessing the possible role of brain structure as mediator in the causal link between early-life factors and behavior. The dataset included information on any prenatal and/or perinatal complications, behavioral problems assessed through the CBCL version 6-18 (CBCL/6-18) [50], and brain morphology based on structural magnetic resonance imaging (MRI). An unbiased whole-brain and voxel-based approach was used to map the associations between prenatal and perinatal factors and GMV in clusters that do not necessarily correspond with predefined region of interest (ROI) boundaries. The resulting brain clusters were selected as ROIs for the subsequent brain-behavioral and mediation analyses. We hypothesized not only that brain development is influenced by prenatal and perinatal factors, but also that brain structural features might act as mediators of the effects of these factors on behavior in the delicate developmental age.

## Methods

### Study protocol

This study fits into the longitudinal GENESIS project, which aimed to extensively characterize neurodevelopmental problems and explore protective and risk factors implicated in their onset. The project involved a clinical sample of children and adolescents who were referred for emotional and behavioral problems at the Scientific Institute IRCCS “E. Medea” (Italy). A baseline demographic and clinical assessment was implemented during childhood. A follow-up clinical and neuroimaging assessment was performed after around six years from baseline. Further details on the GENESIS project can be found in [33, 49]. The dataset employed in our study consisted of data on prenatal and perinatal complications retrospectively collected at baseline, and behavioral and neuroimaging data collected at follow-up. The dataset is protected by privacy and will be available from the corresponding author upon reasonable request, after signature of a formal data sharing agreement.

### Participants

The dataset analyzed in our study was collected from 82 participants of the GENESIS project (28 females, 54 males, 10.07 ± 2.17 years at baseline, 16.58 ± 2.22 years at follow-up). Four centers of the IRCCS “E. Medea” located in Bosisio Parini, Conegliano, Pasian di Prato, and San Vito al Tagliamento were involved in the sample recruitment. Exclusion criteria were diagnoses of autism spectrum disorder, intellectual disability, or neurological diseases (including epilepsy and traumatic brain injuries), severe sensory deficits (i.e., hypo-acusia or visual impairment), and severe linguistic comprehension deficits. The study protocol was approved by the competent Research Ethics Committees and performed in conformity with the Declaration of Helsinki and Ethics Committee guidelines. For all participants, a written informed consent to the protocol was obtained by their parents.

### Clinical assessment

At baseline, the parents retrospectively referred to the occurrence of different types of obstetric complications, either prenatal or perinatal, by responding to a dedicated checklist. Different possible complications were assessed, whose prevalence in the sample is indicated in Table 1. These items were grouped into three composite measures referring to pregnancy, fetal, and delivery complications, which were used for the subsequent analyses. Delivery complications were considered as a separate entity due to their specific time of occurrence, whereas pregnancy and fetal complications were distinguished for their maternal and fetal origins, respectively. This choice is supported by the recent literature on obstetric complications. (e.g., [51]).

**Table 1:** Prevalence of obstetric complications.

Time	Type	Complication	Absolute	With multiple	With single
Pre	Pregnancy	Risk of miscarriage	19 (23%)	13 (15%)	6 (7%)
Pre	Pregnancy	Preeclampsia	4 (5%)	4 (5%)	0 (0%)
Pre	Pregnancy	Rhesus incompatibility	7 (8%)	5 (6%)	2 (2%)
Pre	Pregnancy	Rubella or syphilis	2 (2%)	2 (2%)	0 (0%)
Pre	Pregnancy	Herpes simplex	1 (1%)	1 (1%)	0 (0%)
Pre	Pregnancy	Toxoplasma gondii	1 (1%)	1 (1%)	0 (0%)
Pre	Pregnancy	Other prenatal viral infection	0 (0%)	0 (0%)	0 (0%)
Pre	Pregnancy	Influenza	5 (6%)	2 (2%)	0 (0%)
Pre	Fetal	Preterm birth (<37 weeks)	10 (12%)	7 (8%)	3 (4%)
Pre	Fetal	Retarded delivery (>weeks)	8 (9%)	7 (8%)	1 (1%)
Pre	Fetal	Low birth weight (<2 kg)	3 (4%)	2 (2%)	1 (1%)
Pre	Fetal	Visible physical abnormalities in child	3 (4%)	3 (4%)	0 (0%)
Peri	Delivery	Complicated labor	3 (4%)	3 (4%)	0 (0%)
Peri	Delivery	Intensive care/incubator	12 (14%)	11 (13%)	1 (1%)
Peri	Delivery	Delivery with instruments	2 (2%)	2 (2%)	0 (0%)
Peri	Delivery	Prolonged labor	14 (0.17%)	13 (15%)	1 (1%)
Peri	Delivery	Umbilical cord accidents	7 (8%)	6 (7%)	1 (1%)
Peri	Delivery	Irregular delivery	3 (4%)	2 (2%)	1 (1%)
Peri	Delivery	Preterm rupture of membranes	4 (5%)	4 (5%)	0 (0%)

Number of subjects and percentage in the considered group are reported. Prevalence of obstetric complications in the total sample (column “Absolute”), in participants with more than one complication (column “With multiple”), and in participants with only one complication (column “With single”).

*Pre* prenatal, *Peri* perinatal.

At follow-up, the clinical evaluations included the compilation of the CBCL/6-18 by the participants’ parents, whose scores are reported in Table 2. The CBCL/6-18 explores social competence and behavioral problems in children and adolescents in the 6–18 years age range. For each item, the parent had to mark if the relative behavior was not true, sometimes true, or often true for the child. According to the Achenbach System of Empirically Based Assessment, the scale items were scored to obtain eight subscales (anxious/depressed, withdrawn/depressed, somatic complaints, rule-breaking behavior, aggressive behavior, social problems, thought problems, and attention problems). These subscales were further combined in three composite indices, giving information on internalization, externalization, and total problems.

**Tab﻿le 2:** Behavioral characteristics.

Questionnaire item	Score
Total score	56,268 ± 8614
Internalization	57,256 ± 9755
Externalization	53,439 ± 8580
Anxiety	58,024 ± 8224
Withdrawn	58,354 ± 7637
Somatic problems	57,500 ± 7389
Social problems	56,305 ± 6740
Thought problems	55,305 ± 7381
Attention problems	59,780 ± 9835
Rule-breaking behavior	55,244 ± 5160
Aggressive behavior	55,829 ± 6532

*CBCL/6-18* Child Behavior Checklist, version 6–18 years.

Mean ± standard deviation are reported. Description of the CBCL/6-18 scores of the sample.

### MRI data acquisition

The MRI data were acquired at follow-up using 3 T Philips Achieva scanners (Philips, Best, the Netherlands) installed in the University Hospital of Udine (*n* = 43 subjects) and the IRCCS “E.Medea” of Bosisio Parini (*n* = 39 subjects). In both centers, structural brain images were recorded using a T1-weighted magnetization prepared rapid acquisition gradient echo (MPRAGE) 3D turbo field echo (TFE) sequence, with the following parameters: echo time = 3.7 ms, repetition time = 8.1 ms, 190 axial slices with no gap, in-plane field of view = 240 × 240 mm^2^, in-plane matrix size = 240 × 240, isotropic voxel size = 1 mm^3^.

### MRI data processing

The structural MRI dataset was processed on the same workstation using Matlab R2019a (The Mathworks, Inc., Natick) and in-house scripts with functions from the Statistical Parametric Mapping (SPM12, https://www.fil.ion.ucl.ac.uk/spm).

The row T1-weighted images from all participants underwent a first quality check based on visual inspection. The images were manually reoriented as the SPM tissue priors and entered in the SPM12 unified segmentation, which consisted of (i) image correction for intensity inhomogeneity artifacts, (ii) image classification into gray matter (GM), white matter (WM), cerebrospinal fluid (CSF), bone, soft tissue, and air/background, (iii) image registration to tissue probability maps. This step resulted in probabilistic maps for GM and WM tissues in the subject’s native space. For each subject, the GM, WM and CSF tissue class images were summed to estimate the subject’s total intracranial volume (TIV).

Using DARTEL (Diffeomorphic Anatomical Registration Through Exponentiated Lie algebra), the subjects’ GM and WM images were nonlinearly registered and warped into a group tissue map template; the warped GM and WM images were then normalized to the Montreal Neurological Institute (MNI) space, spatially smoothed with a 3D 6 mm full width at half maximum Gaussian kernel to comply with the assumption of normality of model residuals, and modulated to preserve the total amount of signal from each region.

The resulting GM and WM images were subjected to a final quality check using CAT12 (http://www.neuro.uni-jena.de/cat/), which verified the co-registration and intensity homogeneity among the pre-processed images. All GM and WM images passed this quality check. Finally, the SPM masking toolbox was used to extract a group-specific, optimal threshold GM mask that was used as spatial reference for the following voxel-based morphometry (VBM) analysis.

### Statistical analyses

The relationships between clinical and neuroanatomical measures were investigated through multiple linear regression and mediation models using SPM12, the Bootstrap Regression Analysis of Voxelwise Observations (BRAVO) (https://github.com/CoAxLab/BRAVO2), and the Statistics and Machine Learning (https://it.mathworks.com/products/statistics.html) toolboxes in Matlab environment. In the analyses, unilateral interactions were modeled by considering (i) obstetric complications as causal variable, possibly affecting both neuroanatomy and behavior, (ii) neuroanatomy as mediator variable, possibly affected by obstetric complications and in turn affecting behavior, (iii) behavior as outcome variable, possibly affected by both obstetric complications and neuroanatomy. Multiple comparison corrections were performed using approaches adequate to the specific statistical analyses. In the obstetric-brain analyses, where univariate voxel-based statistics were performed, a cluster-based correction was applied. In the other regression and mediation models, Bonferroni corrections were performed by dividing the *p*-value for the number of parallel comparisons. Further details can be found in the dedicated sections.

#### Obstetric complications and neuroanatomy

The effects of obstetric complications on GMV were assessed using a VBM general linear model (GLM) factorial design in SPM12. The design included pregnancy, fetal, and delivery complications as regressors of interest, and age, sex, MRI center, and TIV as additional nuisance covariates. The optimal group-level GM mask was used as an explicit mask for the analyses. After restricted maximum likelihood estimation of the GLM beta coefficients, we inferred on the effects of each type of obstetric complication on voxel-based GMV using positive and negative one-sided *t*-tests, which were the only *t*-tests enabled by SPM12, on the relative GLM beta coefficient (*p* < 0.001). Given the univariate nature of voxel-based statistics, a cluster-based multiple comparison correction was performed by using an arbitrary threshold size of 50 voxels.

#### Obstetric complications and behavior

The association of pregnancy, fetal, and delivery complications with CBCL/6-18 behavioral measures was investigated via GLM analyses using Matlab in-house scripts. Each CBCL/6-18 score was modeled in terms of pregnancy, fetal, and delivery complications as regressors of interest (all in the same model), and age and sex as covariates. The estimation of GLM beta coefficients was followed by inference of the effects of each type of complication on behavior based on two-sided *t*-tests. The significance threshold was set to *p* = 0.05 after correction for multiple comparisons using the Bonferroni method (*N* = 11, i.e., number of parallel comparisons performed, equal to number of CBCL/6-18 scores).

#### Neuroanatomy and behavior

The brain-behavior relationships were investigated in a priori selected phenotypes, that is, by considering the only GM clusters and CBCL/6-18 behavioral measures that resulted significantly influenced by the same obstetric complications (intersection of results from the obstetric-brain and obstetric-behavior analyses). The ROIs were obtained by parceling the VBM clusters using the Automated Anatomical Labeling (AAL) atlas [52]. The GMV within each ROI was estimated by summing the GMV of the corresponding voxels. For each type of complication, we assessed whether the CBCL scores that were affected by the selected complication (if any) were also influenced by the GMV of the ROIs affected by the same complication (if any). To this end, we performed a set of GLM analyses (one for each combination of phenotypes), in which the selected CBCL score was modeled in terms of ROI GMV (regressor of interest, already normalized for TIV), as well as age, sex, MRI center, and presence/absence of any obstetric complications (covariates). The estimation of GLM beta coefficients was followed by inference on the effects of ROI GMV on behavior based on two-sided t-tests. Significance threshold was set to *p* = 0.05 after correction for multiple comparisons using Bonferroni method (*N = *number of parallel comparisons performed, equal to number of CBCL-ROI combinations).

#### Mediation analyses

Any obstetric complication, ROI GMV, and CBCL/6-18 behavioral measures that were found to be mutually associated from the GLM analyses were subject to a mediation analysis, which was aimed to verify whether the impact of obstetric complications on behavior was mediated - and in what proportion - by GMV.

Using the BRAVO toolbox, we designed a simple mediation model on each triad of selected variables, by considering the obstetric complication as the causal variable X, the CBCL/6-18 score as the outcome Y, and the ROI GMV as the mediator M. Provided that X significantly accounts for variability in Y (path *c*) and M (path *a*), and M accounts for variability in Y when covarying for X (path *b*), the mediation model states that M is the mediator of the X-Y relationship if the effect of X on Y substantially decreases when M is entered simultaneously with X as a predictor of Y (path *c’*) (for further details, please refer to ref. [53]).

In our study, we used a mediation regression model with age, sex, and MRI center as covariates. The mediation significance was assessed via permutation with 1000 iterations. Before running the analyses, the values of X, Y, and M were normalized using the Z-score standardization. The strength of model coefficients, for both real data and bootstrap distributions, was assessed through ordinary least square regression. The bias corrected and accelerated formula described in ref. [54] was used to estimate confidence intervals and *p*-values. Significance threshold for all model coefficients (*a*, *b*, a*b, *c*, *c’*) was set to *p* = 0.05 after correction for multiple comparisons using Bonferroni method (*N=* number of mediation models fitted).

#### Statistical power analysis

Since our exploratory study was the first to investigate the mutual relationships among obstetric complications, brain, and behavior, no prior scientific knowledge was available to estimate a formal sample size for our analysis. Nevertheless, a power analysis for causal mediation models was implemented [55] by hypothesizing the following: 30% of probability of any obstetric complications, *a* and *c* coefficients equal to 0.4, *b* coefficient equal to 0.3, a*b coefficient equal to 0.1, and Y’s and M’s variances explained by X equal to 20%. As in our mediation model, three covariates were considered. The Monte Carlo confidence interval method was used for estimation of causal mediation effects. The numbers of replications per sample size for power calculation and of Monte Carlo draws per replication were set at 1000. The random seed was set at 1. The power analysis confirmed that a sample size of 80 individuals was sufficient to estimate the total indirect effect with a statistical power greater than 95% at a significance level of *p* < 0.05.

## Results

The results of the GLM analyses between (i) obstetric complications and neuroanatomy, (ii) obstetric complications and behavior, and (iii) neuroanatomy and behavior, and of the GLM-driven mediation analyses are reported in the following sections.

### GLM analyses

#### Obstetric complications and neuroanatomy

The GLM statistics concerning the effects of each category of obstetric complications on voxel-based GMV are reported in Table 3. Complications during pregnancy, including infections, were associated with reduced GMV in clusters within the left mid occipital cortex, the right superior medial frontal and anterior cingulate cortex, and the right superior frontal gyrus. Fetal complications were associated with greater GMV in portions of the left middle occipital cortex and of the right superior frontal gyrus. Complications during delivery were associated with greater GMV in a portion of the right superior frontal gyrus, and with lower GMV in two clusters located in the left fusiform gyrus and left putamen.

**Table 3:** Voxel based morphometry results.

Complication	AAL atlas regions	# voxels	*p*	peak T	MNI coordinate
Pregnancy	middle occipital cortex, L	56	<0.001	−4.01	−39, −72, 33
	superior medial frontal cortex and anterior cingulate cortex, R	101	<0.001	−3.96	12, 54, 14
	superior frontal cortex, R	71	<0.001	−3.88	24, 45, 38
Fetal	middle occipital cortex, L	168	<0.001	4.03	−26, −98, −2
	superior frontal cortex, R	129	<0.001	3.89	20, 54, 2
Delivery	superior frontal cortex, R	50	<0.001	4.14	27, 5, 66
	fusiform, L	63	<0.001	−3.97	−24, −74, −5
	putamen, L	59	<0.001	−3.64	−20, 3, 9

MNI coordinates: x, y, z coordinates of peak T in mm. L: left hemisphere. R: right hemisphere. VBM statistics regarding the association of obstetric complications and gray matter volumes.

*VBM* voxel-based morphometry, *AAL* Automated Anatomic Labeling, *T* T statistics, p *p* value.

#### Obstetric complications and behavior

The GLM analyses assessing the association of obstetric complications with behavioral measures revealed a significant positive association between complications during pregnancy (including infections) and the CBCL/6-18 withdrawn score (T(76) = 3.02, *p* = 0.003), indicating greater withdrawal issues in the individuals characterized by prenatal maternal complications.

#### Neuroanatomy and behavior

Based on the above results, we investigated brain-behavior relationships in the brain regions and behavioral scores that were found to be affected by the same obstetric complications. Specifically, in 6 separate GLM analyses, we assessed the relationships between the 6 ROI GMV and the CBCL/6-18 withdrawn score being affected by pregnancy complications (*p* < 0.05, Bonferroni corrected with *N* = 6). We found an inverse relationship between GMV in (i) the right superior medial frontal gyrus (T(76) = 3.41, p_unc_ = 0.001, p_Bonf_ < 0.05) and (ii) the right anterior cingulate cortex (T(76) = 3.20, p_unc_ = 0.002, p_Bonf_ < 0.05) and the CBCL/6-18 withdrawn score.

### Mediation analyses

**Fig. 1:**
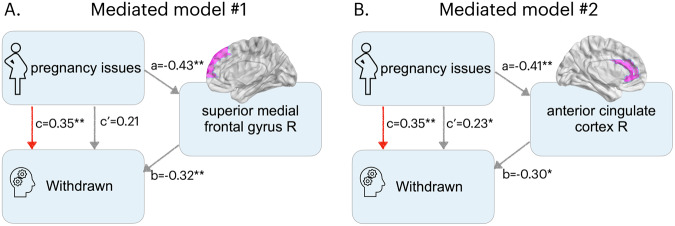
Mediation model results. Estimated coefficients for mediation models investigating direct and indirect effects of pregnancy complications on withdrawn behavior mediated by gray matter volume in the right superior medial frontal gyrus (**A**, mediated model #1) and in the right anterior cingulate cortex (**B**, mediated model #2). c: total effect of pregnancy complications on withdrawn. c’: direct effect of pregnancy complications on withdrawn. a: direct effect of pregnancy complications on gray matter volume. b: direct effect of gray matter volume on withdrawn. *: p_Bonf_ < 0.05 (Bonferroni corrected, *N* = 2). **: p_Bonf_ < 0.01 (Bonferroni corrected, *N* = 2). R: right hemisphere.

In view of the GLM results, two separate mediation analyses (MA1 and MA2) were performed to investigate the relationship among pregnancy complications (causal variable X), CBCL/6-18 withdrawn score (outcome Y) and GMV in (i) right superior medial frontal gyrus (mediator variable M1), (ii) right anterior cingulate cortex (mediator variable M2). Thus, statistical significance was assessed using Bonferroni correction with *N* = 2. The mediation analysis findings are illustrated in Fig. 1.

In line with GLM results, both mediation analyses confirmed a significant total association of pregnancy complications with CBCL/6-18 withdrawn score (*c* = 0.35, p_unc_ = 0.001, p_Bonf_ < 0.01).

MA1 results confirmed that complications during pregnancy were associated with lower GMV in the right superior medial frontal gyrus (*a* = −0.43, p_unc_ < 0.001, p_Bonf_ < 0.01), and in turn that such GMV deficits (while regressing out the effect of complications) were associated with CBCL/6-18 withdrawn score (*b* = 0.32, p_unc_ < 0.001, p_Bonf_ < 0.01). After the inclusion of M1 in the model, the direct effect of complications on CBCL/6-18 withdrawn score was not anymore significant (*c'* = 0.21, p_unc_ = 0.03, p_Bonf_ > 0.05), whereas their indirect effect through GMV in the right superior medial frontal gyrus was significant (a*b = 0.14, p_unc_ < 0.001, p_Bonf_ < 0.01). Specifically, 39.50% of the total effect of complications on CBCL/6-18 withdrawn score was mediated by GMV in this region.

Similarly, MA2 results confirmed the associations between pregnancy complications and lower GMV in the right anterior cingulate cortex (*a* = −0.41, p_unc_ < 0.001, p_Bonf_ < 0.01), and between the latter and CBCL/6-18 withdrawn score while controlling for complications (*b* = 0.30, p_unc_ = 0.007, p_Bonf_ < 0.05). After the inclusion of M1 in the model, the effects of complications on CBCL/6-18 withdrawn score remained significant, both the direct ones (*c'* = 0.23, p_unc_ = 0.02, p_Bonf_ < 0.05) and the indirect ones mediated by GMV in the right anterior cingulate cortex (a*b = 0.12, p_unc_ < 0.001, p_Bonf_ < 0.01). Specifically, 33.86% of the total effect of complications on CBCL/6-18 withdrawn score was mediated by the ROI GMV.

## Discussion

In the present study, for the first time, we explored the impact of prenatal and perinatal factors on behavioral traits within the delicate developmental phase, by posing special attention to the potential mediator role of brain anatomical features.

Our findings suggest a key role of obstetric complications in affecting both brain structure and behavior. Specifically, we found a link between complications during pregnancy, including infections, GMV alterations in the right frontal gyrus, and withdrawn problems. These reciprocal associations were further explored via mediation models, providing valuable information that could be used for developing innovative strategies for psychiatric risk monitoring and prevention of transition from at-risk to disease states.

From a psychopathological perspective, we found that pregnancy complications were associated with higher withdrawn problems. From a neuroanatomical point of view, we observed associations between pregnancy complications and GMV reductions in the right superior medial frontal and anterior cingulate cortices. Finally, for the first time to the best of our knowledge, a mediation effect of the above neuroanatomical features on the influence of prenatal maternal complications and the withdrawn behavioral dimension was identified.

Overall, these results confirm the importance of the early management of infections and other morbidities during pregnancy, given their critical role in brain development and, consequently, in behavioral problems during childhood and adolescence periods [56].

### Pregnancy complications and brain anatomy

Our results suggest a critical role of maternal infections (like rubella, herpes, and toxoplasma) and other health problems (e.g., preeclampsia) in brain development, exerting a long-term impact on brain characteristics. Specifically, critical areas contributing to superior cognitive functions, such as the right superior frontal gyrus and anterior cingulate cortex, resulted to be selectively affected by pregnancy, fetal, and delivery complications.

In our sample, the brain region affected by different types of obstetric complications was the superior frontal gyrus, which is known to be implicated in various superior cognitive processes, including mood, theory of mind, and self-awareness, and its alterations have been widely associated with psychiatric disorders [57, 58].

In our sample, complications during pregnancy were associated with lower GMV in the anterior cingulate cortex, which is involved in motivation, decision making, learning, cost-benefit calculation, conflict, and error monitoring as well as social decision making [59, 60]. The anterior cingulate cortex seems also implicated in empathy and prosocial behavior [61] and is altered in different psychiatric conditions such as depression [62–64] and bipolar disorder in adults [65, 66] and adolescents [64, 67, 68]. Therefore, it is not surprising that in our sample GMV deficits in this region corresponded to greater withdrawn behavior, since this trait has been linked in previous studies to an altered volume and function in these structures [69–71].

The existence of a link between obstetric complications and frontal brain morphology is supported by a vast literature evidence. Multiple studies agreed on the existence of frontal and cognitive abnormalities in individuals with very low birth weight [72]. Moreover, our results are in line with human and animal studies suggesting that infections during pregnancy might influence later brain development [73]. Specifically, MRI studies in both the prenatal and the postnatal periods detected alterations ranging from microcephaly, cystic evolution calcifications in the cortico-subcortical junction, frontal polymicrogyria, ventriculomegaly and extensive delayed myelination, also in frontal lobes, associated with infections during pregnancy [74, 75]. Also, in a sample of preterm infants, infections during pregnancy were associated with alterations in both GMV and WMV in the left frontal lobe [76]. Similarly, animal models showed that maternal immune activation after infection during pregnancy was associated with impairments in tasks dependent on the prefrontal cortex and schizophrenia-like phenotype in the offspring [77]. Moreover, a recent study [78] suggested that maternal immune activation might alter the expression of genes involved in neurodevelopment (e.g., genes involved in glutamatergic neurotransmission, mTOR signaling, and potassium ion channel activity) and might have a negative impact on social behavior of the offspring, thus creating an autism-like phenotype. More generally, prenatal complications seem to result in a wide spectrum of mental illnesses ranging from psychotic disorders to depression and anxiety in adulthood [3]. In this regard, prenatal infections and inflammation seem to play an important role in schizophrenia [79] and a large body of evidence sustain the hypothesis that prenatal exposure to infections may cause changes in fetal brain development that further lead to a wide spectrum of cognitive deficits and neuropsychiatric disorders, including autism spectrum disorder, schizophrenia, depression, and bipolar disorder [56, 73, 80].

### Pregnancy complications and behavior

Our findings suggest that complications during pregnancy might affect developmental behavior in the specific withdrawn dimension, leading to a higher score in the corresponding CBCL/6-18 subscale. This result is in line with existing evidence reporting an association between obstetric complications, including infections, and behavioral issues in children [29–32].

Indeed, internalizing behavior in offspring has been linked to prenatal maternal infection/immune activation [32]. Furthermore, interleukin levels and maternal inflammation during pregnancy were found predictive of more severe internalizing symptoms, especially in female offspring [81]. Similarly, maternal infections during the second trimester were significantly associated with depression during adolescence [82].

Regarding the CBCL/6-18, withdrawn behavior has been found to anticipate the development of numerous behavioral and psychiatric disorders, especially mood disorders [41, 83, 84]. Since children with sub-clinical elevations in withdrawn scores have an increased risk of developing depression in the future [84], the withdrawn score elevation can be considered a clinical risk indicator for future depression.

However, why maternal immune activation can lead to alterations in offspring’s behavior is not clear yet. One of the proposed biological theories suggests that immune activation in a critical development period could alter the hypothalamus-pituitary axis, with consequent worse stress response, ultimately leading to depression and anxiety [3, 85].

Therefore, given the renowned association of withdrawn problems in childhood and adolescence with the future development of psychopathology [83, 86, 87], our and previous evidence remarks the need to prevent any infections during pregnancy. Indeed, in a cohort of more than 1 million children, the risk of psychiatric disorders in both childhood and adolescence was found to increase in a dose-response fashion with the number of maternal infections [88].

Conversely, our sample showed no influences of fetal complications on behavioral traits. This negative finding seems in contrast with previous knowledge of long-term effects of preterm birth and low birth weight on the onset of psychiatric disorders [9, 89–91]. This inconsistency might be due to the peculiar characteristics of our study, including the consideration of behavioral traits as a continuum instead of psychiatric diagnoses and the merging of different fetal complications into one category.

### Neuroanatomy and behavior

In our study, we found a negative association between the withdrawn score of the CBCL/6-18 and GMV in the frontal brain regions that resulted influenced by prenatal maternal factors, suggesting a relationship between these three components.

Previous neuroimaging studies performed in similar samples report widespread brain alterations associated with depressive and internalizing symptoms, encompassing both cortical and subcortical structures [69, 92–95]. In line with our findings, in a study conducted on children with a history of traumatic brain injury, right frontal cortical thickness was predictive of social problems [92]. Similarly, the left frontal cortex was associated with worse internalizing problems in children with intractable epilepsy [93]. Of note, in a recent mediation analysis [96], frontal gyrus volume was reported to significantly mediate the effect of adolescent-maternal relationship quality on both internalizing symptoms and adaptive functioning.

Similarly, functional studies confirmed the putative association between frontal brain areas and internalizing symptoms. In a study conducted on adolescents, resting-state functional connectivity between amygdala and superior frontal gyrus was associated with higher internalizing symptoms [97]. Altered amygdala connections with different cortical areas including the cingulate cortex have also been associated with social and emotional impairment in a sample of prematurely born [69], suggesting a role of this circuit in developing internalizing symptoms.

### Role of frontal neuroanatomy in mediating the relationship between prenatal complications and withdrawn behavior

The mediation analyses performed in our study suggested that neuroanatomy acts as a mediator in the association between obstetric complications and withdrawn scores measured via the CBCL/6-18. To our knowledge, this is the first evidence of a possible mediator role of neuroanatomy in the developmental pathway from maternal infections and morbidities during pregnancy to internalizing symptoms during adolescence. This finding could shed light on the underlying mechanism of the developmental origin of adults’ disease (also known as the fetal origin hypothesis) according to which many adult diseases find their basis in prenatal exposure [98, 99].

Although novel, these findings are consistent with the extant literature on the functions of the frontal cortex, highlighting its role in emotion regulation, which typically develops in adolescence [100]. However, the mechanism underlying the mediation effect of the frontal cortex is still unknown. One of the possible putative causes behind the relation between maternal risk factors and withdrawn behavior in children is the dysregulation of the inflammatory response, including proinflammatory molecules [101]. Immune activation in critical developmental periods could result in alteration in the hypothalamus-pituitary axis, with consequent worse stress response [5, 85]. Similarly, perinatal stress might have an impact on the connection between the amygdala and frontal brain areas [102], thus leading to further alterations during adolescence [69]. Given the cruciality of the adolescent period and the key role of the amygdala–prefrontal circuitry in emotional self-regulation, an alteration in this pathway could reflect an initial vulnerability to internalizing symptoms [69, 97] and further risk to develop anxiety or depression [103].

In this context, our results could help the study of intermediate phenotypes or endophenotypes that might clarify the etiology of otherwise complex syndromes. Endophenotypes have only minimally been utilized to explore the perinatal development of internalizing symptoms and psychiatric vulnerability [104]. Regarding the risk of depression, frontal regions have been reported as putative areas that might be regarded as endophenotypes for the development of such psychiatric disorder [105].

However, more research is needed to clarify the effect of prenatal stressors on brain development and complex behaviors in adolescents and adults, particularly because our analysis included only one behavioral and MRI assessment. Nonetheless, in line with the gene-environment interaction model [106], our result points out the fundamental importance of studying the interplay between environmental risk and protective factors and both brain structure and behavior. By unveiling endophenotypes that might further allow us to better define and stratify behavioral risk, it would be possible to create specific interventions to be conducted in time windows that anticipate the transition from the at-risk state to the onset of disabling psychiatric disorders.

### Limitations and conclusions

Despite its potentially relevant contributions to the neuroscientific literature, this study has limitations that should be considered when interpreting the results. First, the sample size is limited by difficulties in conducting the MRI acquisitions, which required a high level of participants’ compliance that was not always achieved due to the young age and clinical characteristics of our sample. Consequently, our sample size is on the low end of what is recommended for moderated mediation models [107]; our results should, therefore, be considered as a preliminary evidence suggestive of a mediation role of brain anatomy on the influence of prenatal issues on internalizing behaviors. However, our sample characteristics are in line with those from other studies in the field [96, 108]. The imbalance between females and males represents a confounder that might have affected the results even after the inclusion of sex as biological covariate in the analyses. Moreover, a limit to our analysis is given by the fact that, luckily, prenatal and perinatal complications are not common events, as they are reported in a minority of our sample. Therefore, larger samples are needed to obtain higher statistical power and our results should be tested on bigger replication samples to minimize the risk of false positives; multicentric approaches could reduce the possible sources of bias due to other environmental confounders, such as ethnicity, social environment, and other stressors acting during infancy and adolescence. In our statistical analyses, the consideration of age and sex (and MRI scanner and TIV in the GMV analyses) as the only covariates might have left residual confounders like postnatal environmental factors (maternal mental health, discrimination, school environment, social protective factors, etc.), which should be taken into account in future analyses. Lastly, our regression and mediation models inevitably approximated the complex relationships among early environmental factors, brain, and behavioral traits. A unilateral brain-behavior relationship was assumed, in which internalizing symptoms represented the phenotype (outcome) partly affected by brain morphology (mediator). Although behavioral influences on the brain could not be excluded, we hypothesized them to be minor and time-delayed compared to brain influences on behavior. Future works should more deeply investigate the complex brain-behavior relationships and employ mediation models accommodating their bilateral interactions.

Future studies should also include other behavioral measures, information on cognitive performances, and multimodal neuroimaging techniques to improve our understanding on the pathways leading from prenatal and perinatal disturbances to altered behaviors. As a future perspective, newer strategies, such as fetal MRI in combination with new genetic methods, could be useful in detecting even earlier structural alterations and to follow-up patients with multiple scans in different periods of life.

In conclusion, our findings suggest a key role of obstetric complications in affecting both brain structure and behavior in children and adolescents. Thanks to the use of a mediation design, we provided evidence for a mediator role of frontal cortex anatomy in the relationship between prenatal maternal issues and internalizing symptoms. Nonetheless, it would be desirable to promote prevention and early-treatment programs for health during pregnancy, which we believe to be a primary goal of all care plans. Furthermore, early behavioral assessments in childhood should be encouraged to address in such a crucial time window the risk factors for the future development of disabling psychiatric disorders.

## Data Availability

The clinical and MRI data supporting the study have not been deposited in a public repository because of privacy and ethical restrictions, but are available from the corresponding author upon reasonable request, after signature of a formal data sharing agreement. Matlab codes used for data analysis are available at the Opens Science Framework web application link (https://osf.io/yebtp/, 10.17605/OSF.IO/YEBTP).
